# Coarse Eyeball Direction Recognition from Eyelid Skin Deformation Using Infrared Distance Sensors on Eyewear

**DOI:** 10.3390/s26092636

**Published:** 2026-04-24

**Authors:** Kyosuke Futami

**Affiliations:** 1 Graduate School of Information Science and Engineering, Ritsumeikan University, 2-150 Iwakuracho, Ibarakishi 567-8570, Japan; futami@fc.ritsumei.ac.jp; 2 Digital Spirit Tech, Ibarakishi 567-8570, Japan

**Keywords:** eyewear, eye, eyeball direction, gaze, hands-free input, wearable device, infrared distance sensor

## Abstract

As smart eyewear becomes increasingly widespread, the need for hands-free input interfaces is growing. Although eye-based input is a promising approach, many everyday interactions do not necessarily require the high-precision gaze-point estimation used in mainstream camera-based systems; instead, what is often needed is the recognition of coarse eyeball direction. In this study, we propose a method for recognizing coarse eyeball direction using infrared distance sensors mounted on eyewear. The proposed method leverages deformation patterns in the eyelid and surrounding skin associated with changes in eyeball direction. The evaluation results show that the proposed method achieved macro-F1 scores of 0.9 or higher in the best-performing conditions for the five- and nine-direction settings. These results demonstrate the feasibility of recognizing coarse eyeball direction from eyelid-skin deformation using infrared distance sensors on eyewear. Rather than replacing high-precision gaze-point estimation, the proposed method can be positioned as a low-cost, non-contact, and low-dimensional sensing approach for command-type eye-based input on eyewear devices.

## 1. Introduction

In recent years, eyewear-based information devices such as smart eyewear (e.g., AR glasses, audio glasses, and autofocusing glasses) have rapidly proliferated and are expected to become increasingly integrated into everyday life and professional workflows. Providing effective input methods for such devices is therefore a critical challenge. Voice input is often undesirable in public settings, whereas hand gestures and touch interactions on the frame are impractical when the user’s hands are occupied (e.g., while carrying objects or performing manual tasks). In this context, eye-based hands-free input is a promising approach and can provide one of the most natural and immediate forms of interaction for smart eyewear.

However, for many everyday input interactions on smart eyewear (e.g., checking notifications or information, controlling media playback, or unlocking via an eye-movement pattern), the high-precision gaze-point estimation targeted by conventional eye trackers is not necessarily required. In such cases, what is needed is the recognition of coarse eyeball direction (e.g., looking up or looking right), which enables discrete, gesture-like input. Mainstream camera-based eye-tracking techniques can achieve high accuracy, but because they rely on continuous image acquisition and processing, the demands associated with data acquisition and processing (e.g., required processor capability, power consumption, storage requirements, communication volume, long-term data capacity, and latency) tend to increase. Indeed, there is a growing trend toward considering non-imaging, low-dimensional alternative sensing approaches [1,2,3]. For resource-constrained eyewear devices, continuously operating a camera solely to realize simple command-type input is not necessarily a practical design choice for such devices. In addition, privacy concerns arising from the continuous capture of the user’s eyes or face may reduce social acceptability. For these reasons, developing a low-cost sensing method that can recognize coarse eyeball direction without compromising privacy would be beneficial for smart eyewear.

In contrast, there are approaches that use infrared (IR) distance sensors mounted on eyewear to continuously sense eyelid-skin movements. For example, such sensors have been used to recognize blinks [4,5], facial expressions [6,7], and eyeball-movement direction [8,9]. Prior work has also shown that IR distance sensors can robustly capture skin motion while requiring relatively low processing power and energy consumption, making them suitable for always-on sensing devices [4,10,11,12]. However, the extent to which this approach can recognize eyeball direction has been only sparsely investigated. Although we expect the directional resolution of our method to be lower than that of camera-based approaches, demonstrating that it can still estimate coarse eyeball direction would provide valuable insights.

In this study, we propose a method for recognizing coarse eyeball direction using infrared distance sensors for simple eye-based input on eyewear devices. We focus on changes in skin shape associated with eyeball direction. The shape of the eyeball is subtly reflected in the eyelids and surrounding skin. In the proposed method, these skin-surface displacements are measured using an infrared distance sensor array placed on the inner side of an eyewear frame, and the eyeball direction is estimated by machine learning. If coarse eyeball direction can be recognized using only the acquisition and processing of low-dimensional data from infrared distance sensors, this approach could serve as a camera-free eye-based input system for eyewear devices. To demonstrate the feasibility of the proposed method, we implemented a prototype system and conducted evaluation experiments.

The contributions of this study are the following points. First, we propose a method for recognizing the coarse eyeball direction required for simple eye-based input on eyewear devices through non-contact measurement of eyelid skin shape using infrared distance sensors. Second, we implement a prototype system and demonstrate its feasibility experimentally. The key insight underlying this method is that the skin around the eyelid deforms in direction-dependent patterns, and that these patterns can be identified from low-dimensional distance signals.

In this paper, eyeball direction refers to the coarse direction of the eyeball relative to the eyewear or head, rather than precise gaze-point estimation or explicit target-of-attention inference. Although related studies sometimes use the term gaze direction for similar phenomena, we adopt eyeball direction to avoid ambiguity and to emphasize that our method targets the current directional state of the eyeball itself. Related work sometimes uses the term gaze direction in a broader sense, including the rotational state of the eyes with respect to the head. In contrast, this paper intentionally uses eyeball direction to denote that narrower concept explicitly.

## 2. Related Work

### 2.1. Eye Sensing Technologies Integrable into Eyewear

Various sensing modalities have been explored for sensing gaze and eye activity on smart eyewear. With the recent development of diverse eyewear devices, expectations for continuously performing eye sensing on the eyewear itself have been increasing [13].

A major class of such methods is camera-based sensing, which is suitable for high-resolution gaze-point estimation and general-purpose gaze analysis. This category includes methods that combine IR cameras and IR LEDs, as well as methods that use RGB cameras. Commercial products include Pupil Invisible and Neon [14] (Pupil Labs), Tobii Pro Glasses [15] (Tobii), and SMI Eye Tracking Glasses [16]. On the other hand, continuously measuring eye activity by integrating eye cameras into eyewear tends to increase the demands associated with data acquisition and processing, such as required processing capacity, power consumption, storage requirements, communication volume, long-term data capacity, and latency. In addition, these methods raise privacy and security concerns because they capture images of the eyes and face. Even in recent smart-glasses research, it has been pointed out that conventional eye tracking strongly depends on cameras and high computational cost, resulting in “high energy demand” and “privacy issues” [1]. To address these constraints, efforts have also been made to reduce the power consumption of camera-based methods. For example, some low-power and low-latency methods process only locations where changes occur, rather than processing every frame in full, by mimicking information processing in the nervous system [17]. In addition, iShadow and CIDER have been positioned as low-power solutions that achieve both low power consumption and promising accuracy in eyeglass-based settings, and CIDER in particular controls the power–robustness tradeoff by operating in a low-power mode indoors and switching to a more robust model under severe outdoor lighting conditions [18,19]. Furthermore, in recent years, lightweight gaze-estimation frameworks intended for mobile deployment have also been proposed, and reducing computational cost itself has become an important research issue [20]. For this reason, rather than adopting high-resolution gaze estimation as a universal solution for diverse applications, recent work has increasingly considered low-dimensional, low-power, and non-imaging alternative sensing according to the target application [1].

Another approach uses EOG (electrooculogram) sensors on eyewear. This approach places electrodes on the skin near the eyes and senses changes in electrical potential during eyeball movement. The commercial product JINS MEME uses EOG sensors placed on the nose pads and between the eyebrows [21,22,23]. More recently, hybrid EOG systems that combine contact and contactless electrodes have also been proposed for recognizing eyeball movements and blinks in low-power smart-glasses systems [1]. For EOG-based methods as well, electrode miniaturization and reduction in the number of electrodes have long been studied under the assumption of continuous daily use [24]. EOG has also been used in applications such as fatigue estimation by sensing eyelid and eyeball movements [25,26]. While EOG can capture coarse movement directions and event-like changes accompanying eyeball motion, it has limitations in estimating static eyeball direction at high resolution. Because electrooculographic signals arise when the eyeball moves, a signal indicating a specific eyeball direction is not generated simply because the eyeball remains still in that state (e.g., there is no signal that directly indicates that the eyeball is currently facing right). Furthermore, previous studies have reported that recognizing vertical eyeball movement and distinguishing between eyeball movement and eyelid movement are difficult, leaving limitations for high-resolution eye-activity recognition [27,28]. EOG has the advantage of being less affected by ambient light conditions, but it also has challenges specific to contact-type sensors (e.g., skin irritation or itching, and performance degradation due to sweating).

Acoustic sensing has also been investigated. For example, GazeTrak estimates reflected acoustic patterns specific to eyelid skin shape using a speaker and microphones mounted on smart glasses [2]. This study continuously estimates gaze position through acoustic sensing on the eyeglass frame and can be regarded as recent work on low-power gaze estimation. Compared with camera-based methods, acoustic sensing can reduce privacy concerns in that it does not continuously acquire images of the eyes or face. On the other hand, as long as microphones are used, privacy and security concerns related to audio remain, such as the identification or reconstruction of oneself, others, or surrounding situations from speech or environmental sounds. In addition, because prior studies acquire and process data at 50,000 samples per second per microphone, the data acquisition and processing load tends to be higher than that of other non-imaging methods. Furthermore, although this approach is not affected by ambient light in the same way as optical sensors, it may, in principle, also be influenced by external sounds, such as high-frequency components in environmental sounds, whether naturally occurring or intentionally introduced maliciously.

There are also non-imaging optical sensors. This category acquires information related to the eyeball and pupil by irradiating the periocular region with infrared light without using a camera. Broadly, this category includes methods that read reflected-light distributions using IR LEDs and photodetectors, and methods that read back-reflected light interference using laser feedback interferometry. For example, Battery-Free Eye Tracker on Glasses estimates pupil position and pupil diameter using NIR lights and photodiodes, and is a representative example of non-imaging optical sensing using IR light [29]. In addition, methods have been proposed that integrate photodetectors into eyeglasses in an unobtrusive form [3]. Furthermore, there is a method that integrates IR light sensing into the edges of lenses and uses changes in the received-light distribution accompanying eyeball movement to make eye tracking visually unobtrusive [30]. In contrast, methods based on laser feedback interferometry use interference changes generated when laser light reflected by the target re-enters the emitting device, rather than reflected-light intensity itself [31]. Such methods can reduce data volume and computational cost compared with camera-based methods. However, because recognition depends on the distribution of reflected light, they are susceptible to wearing-position shifts, direct sunlight, and individual differences. Recognition accuracy may also decrease for users, or in situations in which part of the pupil or iris contour is occluded by the eyelid, for example, when the degree of eye opening is small.

There are also contact-type methods that measure skin shape and movement using sensors directly attached to the skin. For example, skin-attachable strain sensors have been proposed, and designs aiming to achieve stretchability, linearity, and high sensitivity have been reported [32]. An advantage of contact methods is strong mechanical coupling with the skin, which makes it easier to measure minute deformations. Although such methods differ from the eyeglass-type, non-contact eyeball direction recognition targeted in this study, it has also been shown that sensors attached to the lower eyelid may detect deformation accompanying eyeball movement [33]. On the other hand, contact methods also face issues such as day-long adhesive stability, the effects of sweat, skin irritation, and comfort during long-term use. A recent review of skin-mounted electronics emphasized adhesiveness, breathability, and mechanoelectrical stability as requirements for long-term continuous monitoring, and identified the incompleteness of the skin–device interface and insufficient breathability as important issues [34]. In addition, although not intended for eyeball direction recognition, a related example of integrating contact sensors into eyeglass-type devices has been reported for blink-pattern measurement by capturing skin-pressure changes derived from the orbicularis oculi muscle using a flexible pressure sensor [35].

As a non-contact method for measuring skin shape and movement, infrared distance sensors mounted on eyewear have also been studied. This approach recognizes eye activity from changes in the distance between the infrared distance sensor and the skin. It has also been shown that this type of sensor may be suitable for always-on wearable devices from viewpoints including power consumption [4]. Previous studies have shown that intentional eyeball-movement direction can be recognized [8,9], and that the same sensing principle can also be applied to blink recognition [4,36]. As a non-contact method, it can reduce issues specific to skin-attached contact-type sensors. Because this method does not directly measure the pupil, iris, or sclera, it may, in principle, still function even for individuals or in situations where part of these structures is occluded by the eyelid, for example, when the degree of eye opening is small. On the other hand, studies that systematically show to what extent the static orientation of the eyeball can be stably recognized remain limited.

Table 1 provides an overview of the eyewear-integrable sensing modalities for eyeball direction recognition. Importantly, the use cases of eye sensing are not uniform. Many camera-based systems mainly target applications that estimate gaze points and fixation positions on a screen as continuous values with high precision. In contrast, in smart eyewear input, there are many situations in which highly accurate gaze-point estimation is not necessarily required, and it is sufficient if coarse eyeball direction can be identified. Because different sensing methods have different characteristics and constraints, they should be selected according to the use case. Therefore, this study does not claim that the proposed method is uniformly superior to existing methods in general. Rather, it shows that a method that measures eyelid skin shape in a non-contact manner using inexpensive distance sensors can be a promising option for coarse eyeball direction recognition under the constraints of eyewear devices. It is meaningful to increase the options of sensing methods with different characteristics.

### 2.2. Recognition Methods of Skin Movements Using Infrared Distance Sensors

Skin-movement sensing using infrared distance sensors has been applied to various wearable interfaces. In the facial area, prior studies have used deformations around the cheeks as an input source and treated cheek-rubbing motions and contact operations on the cheek surface as gestures [37,38], showing that infrared distance sensors can serve as stable input signals for local facial-skin movements. Infrared distance sensors have also been used to recognize silent speech in eyewear- and ear-hook-type wearable systems [12,39]. In addition, studies have been reported that recognize smiles by acquiring skin deformations around the cheeks and the outer corners of the eyes [7], and that identify multiple facial expressions, such as smiles and surprise, in daily situations by using movements of the eyelids and cheeks [10]. Furthermore, a method has been proposed in which infrared distance sensors mounted on a head-mounted display reflect the user’s facial skin movements in avatar facial expressions in a virtual space, showing that minute deformation information from the user’s body can be connected to real-time interaction [40].

Infrared distance sensors have also been applied to body parts other than the face. In ear-mounted devices, input methods have been proposed that recognize facial gestures [41] and detect ear-pulling motions and tongue movements [42,43]. As an example of extending the target body parts to the oral cavity, fingers, and limbs, tongue gesture recognition using a mouthpiece has also been reported [44]. Recognition of finger orientation and movement using ring-type devices [11], as well as hand shape gesture recognition using wristband-type devices [45], has also been reported. In addition, there have been attempts to obtain eyeball movements from infrared distance sensors placed inside an eye mask and to apply them to sleep quality evaluation [46]. Taken together, these studies indicate that infrared distance sensors can capture a variety of movements of skin and soft tissues depending on the wearing position.

For integration into eyewear, infrared distance sensors have the advantage that they can acquire deformations around the eyes and on the face from inside the frame without requiring direct skin attachment or image acquisition. Therefore, they can be regarded as a promising means of handling skin movements in a low-load and non-contact manner in wearable devices intended for continuous wear. On the other hand, many existing studies have targeted single gestures or facial-expression recognition at specific body parts, and methods for recognizing eyeball direction from eyelid skin deformation using non-contact sensing integrated into eyewear remain limited. This study addresses this gap by investigating eyeball direction recognition from eyelid-skin deformation using non-contact infrared distance sensors on eyewear.

## 3. Method

In the proposed method, coarse eyeball direction (e.g., up, down, left, and right) is recognized from changes in skin geometry associated with eyeball direction. We assume that the shape and motion of the eyeball are subtly reflected through the eyelids. These skin movements are sensed from changes in the distance between the skin and infrared distance sensors placed in front of the eyes. Infrared distance sensors measure the distance to an object using infrared light.

**Figure 1:**
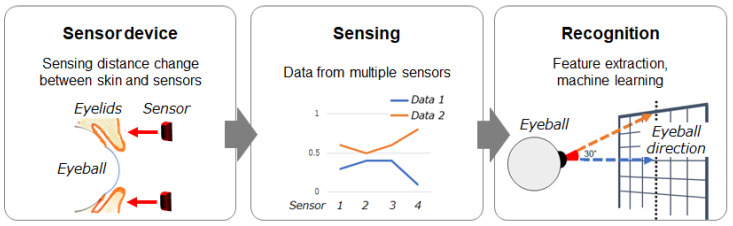
Overall flow of the proposed method.

Figure 1 shows the overall flow of the proposed method. It consists of the following three stages. (1) *Sensing stage*: multiple infrared distance sensors measure skin shape associated with eyeball direction based on changes in the distance between the sensors and the skin. (2) *Data processing stage*: feature vectors are extracted from the obtained sensor data. (3) *Recognition stage*: machine learning is applied to the feature vectors to estimate eyeball direction.

The proposed method uses infrared distance sensors. Previous studies have shown that applying machine learning to data from multiple infrared distance sensors enables robust recognition of various skin movements. Based on this background, we assume that the proposed method can also robustly recognize periocular skin movements associated with eyeball direction.

### 3.1. Data Processing and Recognition Mechanism

In this study, eyeball direction is estimated for each time sample by applying machine learning to a feature vector constructed from the instantaneous sensor values. We assume that information related to eyeball direction is reflected in the instantaneous spatial pattern across the sensors. Two types of feature vectors were used. The first is a 16-dimensional feature vector consisting of the raw values from the 16 infrared distance sensors. The second is a 40-dimensional feature vector constructed from the raw sensor values together with difference features between adjacent and diagonal sensor pairs. This feature construction was adopted based on prior work using an expanded set of spatial-difference features for infrared-distance-sensor-based skin-movement recognition. Three types of machine-learning classifiers were applied to these feature vectors: SVM (support vector machine), Random Forest, and kNN (*k*-nearest neighbor).

### 3.2. Implementation

**Figure 2:**
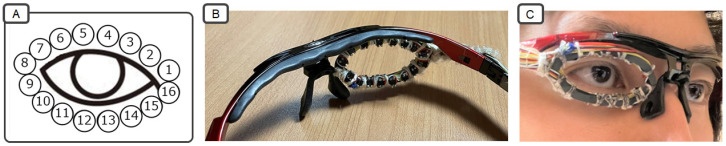
(**A**) Sensor position. (**B**) Sensor device. (**C**) Wearing the sensor device. Adapted from [8], under CC BY 4.0.

**Figure 3:**
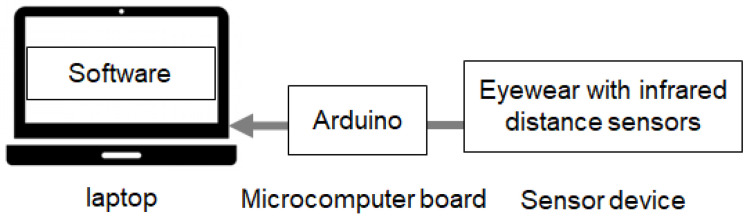
System configuration of the prototype implementation.

We implemented a prototype system based on the proposed method. Figure 2 shows the sensor device. Sixteen sensor positions were arranged on the inner side of the eyeglass frame. Each sensor measured the distance to nearby skin within approximately 1 cm. The overall prototype system consisted of a sensor device, a microcontroller (Arduino), a laptop PC, and software, as shown in Figure 3. The prototype device consisted of 16 infrared distance sensors (TRP-105) mounted on an eyeglass frame. Sensor data were transmitted to the laptop through the microcontroller. The sampling rate was 200 Hz. The software was implemented using Processing (version 4.3) and Python (version 3.11).

## 4. Evaluation 1: Recognition of Eyeball Direction

This experiment evaluated the recognition accuracy of eyeball direction using the proposed method. A total of 14 participants took part in this experiment (mean age: 21 years; range: 20–32 years). This study was approved by the research ethics committee of Ritsumeikan University (2025-087) and was conducted in accordance with the committee’s guidelines.

### 4.1. Eyeball Direction Patterns

**Figure 4:**
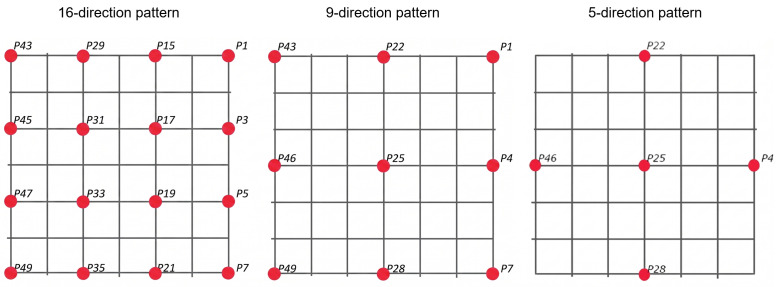
Eyeball direction patterns used in Evaluation 1.

Three eyeball direction patterns were used, as shown in Figure 4. Recognition accuracy was evaluated for each pattern. The reasons for adopting these patterns are as follows.

The 5-direction pattern was used to evaluate the feasibility of an eye-based input interface using the proposed method. One example of a hands-free input application is a media-content player (e.g., for music, video, or still images). For such applications, approximately five commands, such as play, stop, skip forward, and go back, are often sufficient for operation. In fact, the effectiveness of hands-free input methods has been evaluated using approximately five gestures [47,48]. In addition, if five gestures can be recognized, dozens of diverse input patterns can be generated by combining them.

**Figure 5:**
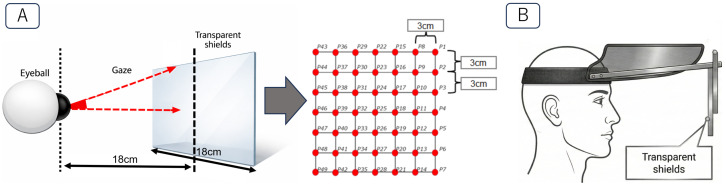
(**A**) Spacing of the target marks used in the eyeball direction task. (**B**) Equipment used to present the target marks in front of the participant’s face.

The 9-direction and 16-direction patterns were used to examine the range and limits of eyeball direction resolution that can be recognized by the proposed method. In addition, if a greater number of directions can be recognized, the range of applicable use cases can be expanded. The corresponding eyeball direction angles were determined by the distance between the eyes and the target points, as shown in Figure 5. Figure 5A shows the spacing of the target marks used in the task. The marks were placed on a transparent shield, and this transparent shield was positioned in front of the participant’s face using a visor, as shown in Figure 5B.

### 4.2. Procedure

The experimental task was a fixation task in which participants gazed at different target points corresponding to different eyeball directions. Each participant wore the prototype device and sat on a chair. In addition, a transparent shield containing the target marks, as shown in Figure 5B, was attached in front of the participant’s face. Target points indicating the required eyeball directions were arranged on the shield.

In one trial, the participant gazed at all target points on the shield. Each point was fixated for 3 s without blinking. This procedure was repeated for three trials. Participants performed the same task for all eyeball direction patterns.

### 4.3. Results and Discussion

**Figure 6:**
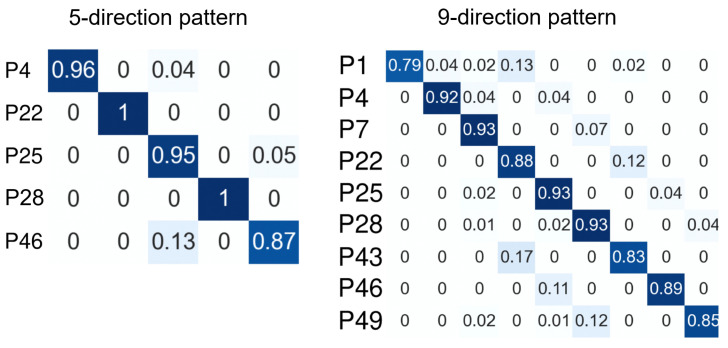
Confusion matrices for the user-dependent five-direction and nine-direction tasks in Evaluation 1. Each matrix summarizes how often each target eyeball direction was classified as each predicted direction. Misclassifications remained limited in the lower-resolution direction sets, indicating that coarse eyeball direction patterns were relatively separable in these conditions.

**Figure 7:**
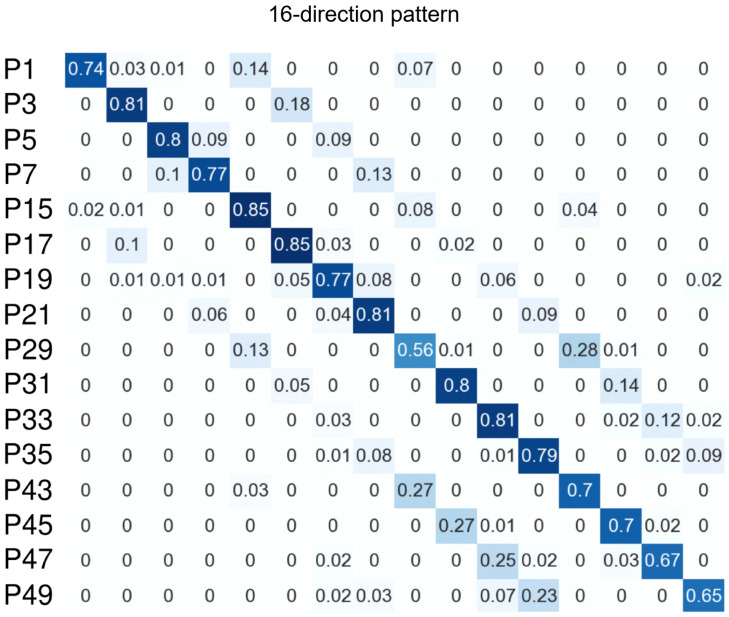
Confusion matrix for the user-dependent 16-direction task in Evaluation 1. As the direction set became denser, errors increased, particularly between neighboring directions, suggesting reduced separability of periocular deformation patterns at higher directional resolution.

Table 2, Table 3 and Table 4 summarize the mean, standard deviation, and 95% confidence intervals for precision, recall, and macro-F1 across participants. The following statistical analyses focus on macro-F1 as the primary performance measure. The 95% confidence intervals of the means were calculated as t-distribution-based confidence intervals. Subject-dependent three-fold cross-validation was performed on the data from the three trials so that data from the same trial were not mixed between the training and test sets. Because multiple combinations of machine-learning classifiers and feature vectors were evaluated, as described in Section 3, hyperparameters were optimized within each training fold. A classifier was then trained using the optimal hyperparameters and the full training data and was evaluated on the corresponding test fold. For the kNN model, *k* was set to 3. Table 5 reports per-participant macro-F1 to illustrate inter-subject variability in the user-dependent setting. Figure 6 and Figure 7 show the confusion matrices for each eyeball direction pattern, which help interpret the tendency of misclassification as the number of target directions increases.

In the statistical analysis, the per-subject macro-F1 score in Evaluation 1 was used as the primary performance measure. Because all conditions were evaluated within the same participants, the data were treated as repeated-measures data. In addition, because macro-F1 is bounded between 0 and 1, we used the aligned rank transform (ART) analysis as a non-parametric factorial analysis corresponding to a three-way repeated-measures ANOVA. The model included classifier type, feature-vector dimensionality, direction resolution, and their interactions. The ART analysis showed a significant main effect of direction resolution (F(2,26)=69.68, p<0.001). The main effect of classifier type was not significant (F(2,26)=2.52, p=0.100), indicating that no statistically significant difference was found among SVM, RF, and kNN. The main effect of feature-vector dimensionality was significant (F(1,13)=20.37, p<0.001). The interaction between feature-vector dimensionality and direction resolution was also significant (F(2,26)=5.78, p=0.008). In contrast, the interaction between classifier type and feature-vector dimensionality (F(2,26)=1.67, p=0.208), the interaction between classifier type and direction resolution (F(4,52)=1.87, p=0.129), and the three-way interaction among classifier type, feature-vector dimensionality, and direction resolution (F(4,52)=2.34, p=0.067) were not significant.

For the significant main effect of direction resolution, post-hoc comparisons were conducted using Wilcoxon signed-rank tests with Bonferroni correction. In the 16-dimensional feature condition, the 5-direction task showed significantly higher macro-F1 than the 9-direction task (p=0.0029), the 5-direction task showed significantly higher macro-F1 than the 16-direction task (p=0.00073), and the 9-direction task showed significantly higher macro-F1 than the 16-direction task (p=0.00073). Similarly, in the 40-dimensional feature condition, the 5-direction task showed significantly higher macro-F1 than the 9-direction task (p=0.0205), the 5-direction task showed significantly higher macro-F1 than the 16-direction task (p=0.00073), and the 9-direction task showed significantly higher macro-F1 than the 16-direction task (p=0.00073).

To interpret the significant interaction between feature-vector dimensionality and direction resolution, we compared the 16-dimensional and 40-dimensional feature vectors within each direction-resolution condition. After Bonferroni correction, no significant differences were found between the 16-dimensional and 40-dimensional feature vectors in any direction-resolution condition. These results indicate that direction resolution was the factor that most strongly affected recognition performance. Macro-F1 decreased significantly as the number of target directions increased from 5 to 9 and 16. No statistically significant difference was found among classifier types. Although the ART analysis also showed a significant main effect of feature-vector dimensionality, this effect should be interpreted with caution because the interaction between feature-vector dimensionality and direction resolution was also significant, whereas the post-hoc comparisons within each direction-resolution condition were not significant after correction.

From a practical perspective, the results support the feasibility of the proposed method for simple command-type eye-based input. Across classifiers and feature-vector conditions, the five-direction task achieved macro-F1 values of approximately 0.94–0.95, and the nine-direction task achieved values of approximately 0.86–0.92. These results suggest that coarse eyeball direction classes can be recognized with relatively high accuracy under the present subject-dependent setting. Because a previous gaze-input interface used a similar five-direction command set [48], the present results suggest that the proposed method could support general command-type gaze input. Because dozens of gaze-input gestures can be generated by combining these five directions, the proposed method could support a variety of input operations. In contrast, the 16-direction task showed lower performance, with macro-F1 values of approximately 0.72–0.77, indicating that recognition becomes more difficult as directional resolution increases.

The confusion matrices in Figure 6 and Figure 7 further support this interpretation. In the five-direction and nine-direction tasks, misclassifications remained relatively limited. In contrast, in the 16-direction task, misclassifications increased particularly between neighboring directions. This tendency suggests that as the angular difference between adjacent target directions becomes smaller, the difference between the corresponding periocular skin-deformation patterns also becomes smaller. Accordingly, the feature distributions of the classes become closer to one another, making discrimination more difficult, particularly between neighboring directions. Thus, the proposed method appears to be better suited to coarse directional input than to finer directional discrimination.

Table 5 indicates that recognition performance also varied across participants. Although many participants achieved high macro-F1 in the five-direction and nine-direction tasks, lower values were observed for some individuals, especially in the higher-resolution conditions. This inter-subject variability suggests that the degree to which eyeball direction-dependent skin deformation appears in the sensor signals differs across individuals. From an application perspective, this result implies that personal calibration and user-specific optimization may be important for stable operation.

Although RF showed the highest or near-highest macro-F1 in several conditions, the main effect of classifier type was not significant. One plausible explanation is that the feature vectors in this study included not only the instantaneous values of multiple sensors, but also local difference features between adjacent and diagonal sensor pairs. Such representations may involve nonlinear interactions among channels and threshold-like local variations, and RF may therefore be relatively well suited to capturing these structures. However, this interpretation is based on the observed results and not on a direct analysis of the model internals.

## 5. Evaluation 2: Device Reattachment

The results of Evaluation 1 supported the feasibility of the proposed method. This section investigates how reattachment affects recognition accuracy. Ten participants took part in this evaluation. After completing Evaluation 1, each participant performed one trial of the same fixation task on a different day. We trained the model using the data from Evaluation 1 (before reattachment) and tested it using the data recorded in Evaluation 2 (after reattachment) to evaluate recognition performance after reattachment.

### Results and Discussion

The recognition results for each eyeball direction pattern are shown in Table 6. It summarizes the mean, standard deviation, and 95% confidence intervals for precision, recall, and macro-F1 across participants. The 95% confidence intervals of the means were calculated as t-distribution-based confidence intervals. SVM was used as the classifier in this evaluation.

Under the reattachment condition, the mean macro-F1 for the five-direction pattern ranged from 0.87 to 0.90 across feature conditions. Although recognition performance decreased after reattachment, relatively high performance was maintained when the model trained before reattachment was applied to data recorded after reattachment. These results suggest that the proposed method has a certain degree of tolerance to physical misalignment caused by reattachment.

At the same time, the observed performance decrease indicates that reattachment can shift the feature space and reduce recognition accuracy. Specifically, in the 16-dimensional SVM condition, the macro-F1 decreased from 0.94 to 0.90 in the 5-direction task, from 0.86 to 0.82 in the 9-direction task, and from 0.72 to 0.70 in the 16-direction task. In the 40-dimensional SVM condition, the corresponding values decreased from 0.94 to 0.87, from 0.89 to 0.80, and from 0.75 to 0.68, respectively. These results suggest that the method has a certain tolerance to reattachment, while also indicating that the effect of reattachment is not negligible.

From a practical perspective, lightweight recalibration after reattachment may be effective. For example, after putting on the device, the user could fixate on a small number of predefined directions, such as up, down, left, right, and center, for a few seconds each. Such a procedure may help compensate for shifts in the feature space while keeping the user burden relatively low.

This reattachment experiment should be interpreted as a preliminary robustness check rather than as a complete sensor-displacement sensitivity test. The physical amount of sensor displacement associated with reattachment was not directly measured. Therefore, the present results do not provide a direct quantitative model of sensitivity to sensor displacement. Rather, they indirectly show how recognition performance changes after actual reattachment in a realistic usage situation.

## 6. General Discussion

Based on the evaluation results, we showed that infrared distance sensors mounted on eyewear can serve as an effective means of recognizing coarse eyeball direction. The proposed method should be interpreted not as a replacement for high-precision gaze-point estimation, but as an approach for low-resolution, command-type eye-based input under the constraints of eyewear devices. In this sense, the method could potentially be integrated not only into smart eyewear such as AR glasses, audio glasses, and autofocusing glasses, but also into everyday eyeglasses.

Although the proposed method has limitations in directional resolution, useful applications remain within the range supported by the present results.

One such application is micro-interaction. For example, simple but frequent operations, such as triaging notifications (e.g., opening details or dismissing alerts) or controlling a media player, could be realized by a small set of eyeball direction commands. Because such tasks do not require fine gaze-point estimation, the proposed method may be sufficient for these low-complexity interactions.

A second possible application is socially unobtrusive input in public settings. For widespread adoption of smart eyewear, input methods that are less conspicuous than voice input or large hand gestures are desirable. Because the proposed method uses only subtle changes in eyeball direction, it could support interactions that are less noticeable to bystanders. For example, a user could send a predefined message during a meeting or advance presentation slides hands-free by using simple eyeball direction commands.

A third possible application is lightweight security based on directional eye-movement patterns. By combining coarse directions, the method can generate a variety of input sequences. For example, a sequence such as “up–up–right–down” could be used as a PIN-like code for unlocking a device or triggering authorization. Compared with finger-based input, such eye-based input may also reduce the risk of shoulder surfing in some situations.

The decrease in performance in the 16-direction condition may be explained by the fact that, as the number of classes increased, the angular difference between adjacent directions became smaller and the corresponding skin-deformation patterns became less distinguishable. The variability observed across participants in the 16-direction results suggests that the proposed sensing principle may also be influenced by subject-specific eyelid characteristics and facial shape. Differences in skin characteristics, such as eyelid laxity and elasticity, may affect how easily deformation caused by eyeball movement can be distinguished. In addition, differences in facial shape (e.g., the periocular region, nasal root, and face width) may affect how the eyewear frame is positioned on the face, and as a result, may change the relative position and distance between the sensors and the skin. Furthermore, differences in the fit between the frame and the skin (e.g., the eyewear being slightly lifted away from the face or shaking during wear) may also affect the stability of the measured signals. These factors may help explain why recognition performance differed across participants. However, these anatomical and fitting-related factors were not directly measured in this study. Therefore, they should not be interpreted as definitive causal factors of the observed performance differences, but rather as plausible explanatory candidates. From a practical standpoint, these results suggest that, rather than assuming uniform training data for all users, it may be more advantageous to introduce user-specific fit adjustment and individual calibration. Therefore, at the present stage, it is more appropriate to position the proposed method not as an interface for high-density direction classification, but as a user-adapted coarse eyeball direction input technique.

From the viewpoint of hardware, the present system should be interpreted not as a hardware-optimized product, but as a proof-of-concept prototype for demonstrating the sensing principle. The current implementation uses 16 infrared distance sensors together with an Arduino and an external laptop PC, and it does not constitute a product-level evaluation of total power consumption, total weight, ergonomic fit during wear, or long-term wearability. Even so, several implications can be discussed at the implementation level. Because the proposed method handles only low-dimensional data, it may allow simplification of the required processing system compared with camera-based methods. In addition, the low-dimensional nature of the sensing signals is suitable for lightweight real-time processing. However, because this study did not quantitatively compare power consumption, weight, or battery requirements with camera-based systems, these points should not be interpreted as quantitatively demonstrated advantages, but rather as potential advantages at the method level. Furthermore, the number of sensors used in the present prototype should not be interpreted as the minimum required configuration. Rather, this configuration was adopted at the initial stage with priority given to periocular spatial coverage. Future work should examine whether the number of sensors can be reduced and whether further miniaturization is possible.

Contact-type sensors can also be used to recognize skin deformation. For example, contact-type skin-mounted wearable sensors have advantages such as being able to easily acquire subtle deformation due to strong mechanical coupling with the skin, and having a stable sensor–skin distance. On the other hand, skin-mounted wearable sensors are generally known to have issues during long-term wear, such as day-long adhesive stability, skin irritation, issues of breathability and skin respiration, the influence of sweat, positional reproducibility at the time of reattachment, and lack of ease of use and removal. In contrast, the proposed non-contact IR distance sensor method does not require direct attachment to the periocular skin and can be integrated into an eyewear form factor. The advantage of the proposed method, therefore, lies not in superior sensitivity over contact-type methods but in combining skin-contact-free operation with eyewear integration. For this reason, we adopted the non-contact approach as the more appropriate option for eye-based input on eyewear devices at the present stage.

## 7. Limitations and Future Work

This study has several limitations, which suggest directions for future work.

First, there are demographic limitations. Because the participant group in this study consisted of young adults, the generalizability of the obtained results is limited. In addition, the signals obtained from infrared distance sensors may vary depending on skin tone, for example, because darker skin may absorb infrared light more strongly. Accordingly, it may become necessary to adjust sensor sensitivity according to skin tone. Age-related eyelid characteristics may also affect the sensing results. In the future, we plan to explicitly evaluate these viewpoints by targeting participant groups with more diverse attributes.

Second, this experiment should be interpreted not as a robustness evaluation for all real-world daily-use situations, but as a controlled laboratory evaluation. The present experiment was a task in which participants intentionally fixed their gaze without blinking (i.e., a gaze-dwelling task), and it demonstrated feasibility for use cases in which gaze input is performed in this way. On the other hand, the present experiment did not evaluate situations involving disturbances throughout daily life, and this remains a future issue. For example, regarding the handling of blinking, since prior studies have shown that blinking can be recognized from infrared distance sensors, the proposed method may be used by implementing specifications such as filtering blink intervals, or, if no blink-filtering function is available, determining gaze input by using as a trigger the condition that the gaze is fixed for a short time while the eyes are open.

Third, in situations involving body vibration, such as walking or head movement, the recognition accuracy of the proposed method may decrease. Therefore, if eyeball direction input is to be used under such conditions, possible countermeasures include adopting an interaction policy in which the user stops before performing gaze input while considering the safety of both the user and nearby others, or introducing a mechanism that compensates for vibration-induced noise using IMUs or related sensors. In this study, we assume a use case in which the user looks at targets on a fixed UI displayed on eyewear. In such a case, even if head orientation changes, the relative eyeball direction with respect to the eyewear remains unchanged, and the proposed method may, therefore, still be usable in principle. However, these mechanisms were neither implemented nor evaluated in this study and should be regarded as future design directions rather than verified solutions.

Fourth, evaluation in this study was performed in a user-dependent manner. Therefore, the current results support the feasibility of user-dependent coarse eyeball direction input, whereas user-independent deployment for unseen users remains a future issue. At the same time, because eyelid skin shape can reasonably be expected to differ across individuals, we assume that practical deployment will rely on user-specific calibration rather than on a single common model for all users. Although practical use would require collecting training data for each target direction in advance, we expect this burden to be limited because calibration can be completed within a few minutes.

Fifth, the current prototype is not a hardware-optimized wearable product. Because the present implementation depends on infrared distance sensors, an Arduino, and an external laptop PC, no direct conclusions can yet be drawn regarding total power consumption, total weight, long-term wearing comfort, or product-level cost. These factors should be directly evaluated in future work after further miniaturization, reduction in the number of sensors, and embedded integration.

Finally, the main purpose of this study was to examine the extent to which periocular eyelid skin deformation associated with eyeball direction can be identified as an instantaneous spatial pattern. For this reason, we first adopted representative classifiers commonly used for low-dimensional feature vectors. SVM, Random Forest, and kNN were selected as representative baselines with different inductive biases so that we could confirm that the proposed sensing principle did not depend on a single model. In addition, the handcrafted feature vectors were designed to explicitly represent local spatial differences between sensors. Through these design choices, we prioritized interpretability and low-latency recognition suitable for command-type input. Although sequence models such as LSTM and temporal CNN are promising future candidates because they can utilize temporal dependencies in continuous sensor sequences, their introduction involves additional design and implementation complexity, including choices regarding time-window length, latency, transition handling, and training complexity. Therefore, the present study focused on feasibility based on instantaneous spatial patterns, whereas time-series modeling remains an important direction for future work.

## 8. Conclusions

This study proposed a method for recognizing coarse eyeball direction from eyelid-skin deformation using infrared distance sensors mounted on eyewear. We implemented a prototype system. The results show that the proposed method achieves an F-value of 0.9 or higher in the 5-direction and 9-direction settings, while performance decreases at higher directional resolutions (16 directions). These findings indicate that the proposed method is suitable for coarse directional classification, while its discriminative ability becomes limited as directional resolution increases. Overall, the results demonstrate the feasibility of recognizing coarse eyeball direction from eyelid-skin deformation using infrared distance sensors on eyewear. Rather than replacing high-precision gaze-point estimation, the proposed method should be positioned as a low-cost, non-contact, and low-dimensional sensing approach for command-type eye-based input.

## Figures and Tables

**Table 1:** Overview of eyewear-integrable sensing modalities for eyeball direction recognition. This table provides a qualitative comparison for ease of understanding, and the details depend on the conditions of each study.

Modality Category	Primary Observed Signal	Contact	Volume of Acquired and Processed Data	Granularity of Eyeball Direction Recognition	Typical Computational Demand	Privacy Risk (Image/Audio Acquisition)
Camera-based methods	pupil/iris/sclera	Non-contact	High	High resolution	High	Visual privacy risk (continuous capture of the eyes and face)
EOG-based methods	Potential changes associated with eye movements	Contact (some systems are hybrid contact/non-contact)	Low	To our knowledge, there have been no reports of static eyeball direction recognition using compact sensors on eyewear.	Low	None
Acoustic sensing methods	Periocular skin shape (Reflected acoustic patterns)	Non-contact	Moderate	Moderate resolution (coarser than camera-based methods)	High	Audio privacy risk (microphone use)
Non-imaging optical sensing methods	Pupil/iris/sclera	Non-contact	Low	Moderate resolution (coarser than camera-based methods)	Low	None
Contact skin-deformation sensing methods	Periocular skin shape (strain and pressure)	Contact	Low	To our knowledge, there have been no reports of static eyeball direction recognition using compact sensors on eyewear.	Low	None
Non-contact infrared-distance-sensor-based skin sensing methods	Periocular skin shape (distance changes relative to the skin)	Non-contact	Low	Low resolution (coarse directions)	Low	None

**Table 2:** Recognition performance of SVM in Evaluation 1. Values are reported as mean ± SD and 95% confidence interval.

Feature	Direction	Metric	Mean ± SD	95% CI
16 dim.	5-dir.	F	0.94±0.08	[0.90,0.99]
		P	0.94±0.09	[0.89,0.99]
		R	0.95±0.06	[0.92,0.99]
	9-dir.	F	0.86±0.14	[0.78,0.94]
		P	0.86±0.15	[0.78,0.95]
		R	0.88±0.12	[0.82,0.95]
	16-dir.	F	0.72±0.20	[0.60,0.83]
		P	0.73±0.20	[0.62,0.85]
		R	0.76±0.18	[0.65,0.86]
40 dim.	5-dir.	F	0.94±0.09	[0.89,0.99]
		P	0.94±0.08	[0.90,0.99]
		R	0.95±0.07	[0.91,0.99]
	9-dir.	F	0.89±0.14	[0.81,0.97]
		P	0.90±0.14	[0.82,0.98]
		R	0.91±0.11	[0.84,0.97]
	16-dir.	F	0.75±0.20	[0.64,0.87]
		P	0.77±0.19	[0.66,0.88]
		R	0.79±0.17	[0.69,0.89]

**Table 3:** Recognition performance of RF in Evaluation 1. Values are reported as mean ± SD and 95% confidence interval.

Feature	Direction	Metric	Mean ± SD	95% CI
16 dim.	5-dir.	F	0.95±0.07	[0.91,0.99]
		P	0.96±0.05	[0.93,0.99]
		R	0.96±0.05	[0.93,0.99]
	9-dir.	F	0.91±0.10	[0.85,0.96]
		P	0.92±0.09	[0.86,0.97]
		R	0.92±0.08	[0.87,0.97]
	16-dir.	F	0.74±0.18	[0.64,0.84]
		P	0.76±0.17	[0.66,0.86]
		R	0.78±0.16	[0.68,0.87]
40 dim.	5-dir.	F	0.95±0.07	[0.91,0.99]
		P	0.95±0.07	[0.91,0.99]
		R	0.96±0.06	[0.93,0.99]
	9-dir.	F	0.92±0.07	[0.87,0.96]
		P	0.92±0.08	[0.87,0.97]
		R	0.93±0.06	[0.90,0.96]
	16-dir.	F	0.77±0.18	[0.67,0.88]
		P	0.79±0.18	[0.69,0.89]
		R	0.80±0.17	[0.71,0.90]

**Table 4:** Recognition performance of kNN in Evaluation 1. Values are reported as mean ± SD and 95% confidence interval.

Feature	Direction	Metric	Mean ± SD	95% CI
16 dim.	5-dir.	F	0.95±0.06	[0.91,0.99]
		P	0.95±0.06	[0.92,0.99]
		R	0.96±0.05	[0.93,0.99]
	9-dir.	F	0.87±0.14	[0.79,0.95]
		P	0.88±0.13	[0.81,0.95]
		R	0.89±0.11	[0.83,0.96]
	16-dir.	F	0.72±0.21	[0.59,0.84]
		P	0.73±0.21	[0.61,0.85]
		R	0.75±0.18	[0.65,0.86]
40 dim.	5-dir.	F	0.95±0.07	[0.91,0.99]
		P	0.95±0.07	[0.91,0.99]
		R	0.96±0.06	[0.93,0.99]
	9-dir.	F	0.90±0.11	[0.84,0.97]
		P	0.91±0.12	[0.85,0.98]
		R	0.92±0.09	[0.87,0.98]
	16-dir.	F	0.74±0.21	[0.62,0.86]
		P	0.75±0.22	[0.63,0.88]
		R	0.78±0.19	[0.67,0.89]

**Table 5:** Per-participant performance of SVM in Evaluation 1.

	5-dir.	9-dir.	16-dir.
Sub.1	1	1	0.94
Sub.2	1	1	0.88
Sub.3	1	1	0.89
Sub.4	1	0.95	0.96
Sub.5	1	0.94	0.82
Sub.6	1	0.81	0.91
Sub.7	1	0.96	0.79
Sub.8	0.98	0.99	0.75
Sub.9	0.92	0.85	0.69
Sub.10	0.91	0.7	0.62
Sub.11	0.91	0.84	0.47
Sub.12	0.73	0.7	0.41
Sub.13	0.91	0.71	0.5
Sub.14	0.82	0.56	0.42
Ave.	0.94	0.86	0.72

**Table 6:** Recognition performance of SVM in Evaluation 2 under the device reattachment condition. Values are reported as mean ± SD and 95% confidence interval.

Feature	Direction	Metric	Mean ± SD	95% CI
16 dim.	5-dir.	F	0.90±0.22	[0.76,1.04]
		P	0.93±0.15	[0.83,1.03]
		R	0.91±0.20	[0.79,1.04]
	9-dir.	F	0.82±0.30	[0.62,1.01]
		P	0.82±0.31	[0.62,1.02]
		R	0.84±0.28	[0.66,1.01]
	16-dir.	F	0.70±0.27	[0.53,0.88]
		P	0.72±0.27	[0.55,0.89]
		R	0.75±0.25	[0.59,0.90]
40 dim.	5-dir.	F	0.87±0.21	[0.73,1.00]
		P	0.92±0.14	[0.83,1.01]
		R	0.88±0.19	[0.76,1.00]
	9-dir.	F	0.80±0.33	[0.59,1.01]
		P	0.83±0.31	[0.63,1.03]
		R	0.82±0.30	[0.63,1.01]
	16-dir.	F	0.68±0.28	[0.51,0.86]
		P	0.69±0.28	[0.52,0.87]
		R	0.72±0.26	[0.56,0.88]

## Data Availability

Data are contained within the article.
